# Factors that influence the burden of the caregiver of cardiovascular patients. A multicenter study

**DOI:** 10.1186/s12889-025-25074-0

**Published:** 2025-11-21

**Authors:** M. S. Palacios-Galvez, E. B. Garcia-Navarro, H. José, M. Giusti, M. Raulinajtys-Grzybeck, L. Sousa, I. E. Vannini, J. Czarnek, A. Ortega-Galan

**Affiliations:** 1 COIDESO, Department of Social Psychology, University of Huelva, Huelva, 21071 Spain; 2 COIDESO. Department of Nursing, University of Huelva, Huelva, 21071 Spain; 3 Escola Superior de Saúde Atlântica, Barcarena, 2730-036 Portugal; 4 Health Sciences Research Unit: Nursing, Coimbra, 3046-8511 Portugal; 5 Department of Experimental and Clinical Medicine, University of Florence, Florence, Italy; 6 Department of Management Accounting, Warsaw School of Economics, Warsaw, Poland; 7 Comprehensive Health Research Centre (CHRC), Lisboa, Portugal; 8 Department of Nursing, University of Huelva, Huelva, 21071 Spain

**Keywords:** Stroke patients, Burden, Family caregivers

## Abstract

**Background:**

Stroke is one of the leading causes of dependency and family burden in Europe. Informal caregivers, mostly women, play a crucial role by providing daily support to stroke survivors, often with limited resources and insufficient institutional backing. This situation negatively affects their physical and mental health, increasing perceived burden and healthcare use. Understanding the factors that shape caregiver burden is essential to design effective interventions that enhance caregiver well-being and improve the quality of care provided.

**Methods:**

A descriptive correlational cross-sectional study was performed, with a convenience sample of family caregivers (*n* = 126) of stroke patients in Italy (26.2%), Poland (23.8%), Portugal (25.4%) and Spain (24.6%). Two-thirds were women. Data were collected using a questionnaire on participants’ characteristics and circumstances and the Zarit Burden Interview (ZBI). The data were analyzed using descriptive statistics, Pearson’s product-moment correlation coefficient and t Student test.

**Results:**

The average burden score on the ZBI was 56.6, representing an intense burden, showing gender differences (women scored higher than men) (*t* = 2.358; *p* = .020), but no differences between countries. A significant positive correlation was observed between burden and use of psychopharmaceuticals (*r* = .230; *p* = .012), increased use of social and health care services (*r* = .257; *p* = .005), and weekly time spent on care (*r* = .261; *p* = .000). A significant negative correlation was observed between burden and physical (*r* = − .366; *p* = .000) and psychological health (*r* = − .537; *p* = .000), quality of the relationship with the person they care for (*r* = − .408; *p* = .000), to find a meaning of care work (*r* = − .312; *p* = .000) and the financial situation (*r* = − .470; *p* = .009). The results of Student’s t-tests showed significant differences in the perceived burden depending on receive (or not) support from persons belonging to public, private or non-profit organizations (*t* = -2.669; *p* = .009), and whether (or not) to perform care voluntarily (*t* = 3.693; *p* = .000). No significant relationships were observed between burden and official recognition of caregiver status, sharing care with other family members or knowing that they can rely on them on an ad hoc basis, or receiving public financial support.

**Conclusion:**

This study shows that family caregivers of people with stroke experience intense levels of burden, influenced by health, social and economic factors across all countries studied. Supporting caregivers requires targeted strategies, including self-care promotion, psychosocial and educational interventions, and stronger support systems. Coordinated action from public, private, and non-profit organizations is essential to reduce caregiver burden and improve both caregiver well-being and the quality of care provided.

**Supplementary Information:**

The online version contains supplementary material available at 10.1186/s12889-025-25074-0.

## Introduction

 Cardiovascular diseases (CVD) represent a significant public health burden in Europe, and their prevalence has raised growing concerns over the past decade. It is estimated that cardiovascular disease causes a total of 4 million deaths in Europe and 1.9 million in the European Union each year, most of them from coronary heart disease (CHD) [1], accounting for 47% of all deaths in Europe and 40% in the European Union [2]. This entails an estimated total cost of cardiovascular disease in Europe of €196 billion per year, approximately 54% of total investment in health, and results in 24% of productivity losses [3]. CVD does not only affect developed countries. As we can see, recent data suggest that the impact of this disease is increasing in non-Western countries. According to recent epidemiological data, CVD is responsible for a substantial proportion of morbidity and mortality across Europe [4], this phenomenon not only imposes a considerable clinical challenge but also generates substantial socio-economic consequences [5].

Stroke has become one of the major public health challenges worldwide — not only because of its immediate impact, but also because of the physical, cognitive, and emotional sequelae it leaves behind, affecting both those who suffer it and those who care for them.

According to the most recent data from the Global Burden of Disease, in 2021 there were approximately 93.8 million people living with stroke-related sequelae and nearly 12 million new cases recorded that same year, clearly illustrating its population-wide impact [6, 7]. Although in some countries age-standardized incidence rates have declined thanks to advances in prevention and treatment, the total number of cases remains alarmingly high. The reason lies in the progressive ageing of the population and overall demographic growth, which sustain — and in some cases even increase — the absolute number of cases [8].

A particularly striking finding emerges when examining a less expected age group: youth. A study focusing on individuals aged 15 to 39 years estimated that, in 2021 alone, there were more than 750,000 age-standardized stroke cases in this population segment. These were accompanied by 8.7 million disability-adjusted life years (DALYs) and over 122,000 deaths attributable to stroke. These are far from negligible figures and compel us to reconsider the perception of stroke as a disease exclusively associated with old age [6, 7].

Furthermore, socioeconomic inequalities add another layer of complexity. While some regions report improvements, others — particularly those with lower levels of development — have experienced worrying increases in age-standardized rates. This disparity highlights that health gains are not equitably distributed and that much work remains to be done to close this gap [9].

According to a recent study [8] on the global burden of disease, it is estimated that cardiovascular diseases contribute significantly to disability adjusted for years of life, making it necessary to implement preventive programs, improving the level of care for patients and providing efficient treatment, especially in regions with lower socioeconomic levels [10]. In addition, an association between sociodemographic factors and the prevalence of CVD has been observed, pointing to disparities that require specific attention [10].

When it comes to stroke, it is not only one of the leading causes of disability worldwide but also imposes a prolonged and highly demanding burden on those who take on the responsibility of providing care. In the European context, this caregiving falls predominantly on families. They are the ones who, through informal care or by hiring non-professional personnel, complement the formal care provided by the health system [11]. This situation exposes a fragile support structure, where caregivers—often lacking specific training or adequate backing—face a daily task that is as essential as it is invisible: sustaining the day-to-day life of a dependent person.

In Europe, the provision of care to dependent patients in their environment is mainly carried out by families (informal care) or people hired by them (formal non-professional care), thus complementing the formal care provided by the state [11]. Informal caregivers play an essential role in supporting patients with cardiovascular problems. Long-term care measures and policies cannot be carried out without taking into account the quantitatively crucial role played by informal caregivers. A multicenter study conducted by several European universities [12] used the European Health Interview Survey (EHIS), the European Quality of Life Survey (EQLS), and the Study on Health and Aging in Europe (SHARE) to measure the prevalence of informal caregivers in the European population and analyze the associated sociodemographic factors. The main results of this study show, predictably, that the proportion of informal caregivers is generally higher among women than among men. However, different studies have divergent prevalence rates by sex [12].

The age range of informal caregivers covers various stages of life, from young people to the elderly, highlighting the generational diversity of those who assume this role. Informal caregivers are usually spouses or children of the dependent person, establishing a direct family bond that influences the nature and intensity of care [13]. The education of caregivers varies, ranging from basic to advanced educational levels. This educational diversity impacts their ability to understand and manage patient health [13].

A high percentage of informal caregivers are employed outside the home, balancing work and care responsibilities. This balance can lead to additional tensions [14]. The combination of employment and informal care can have economic consequences, such as reduced working hours or loss of employment, affecting the financial stability of caregivers. Their role ranges from patient hygiene, medication administration to the provision of emotional support and the management of medical appointments [15, 16]. The burden of care faced by these informal caregivers not only has physical but also mental and emotional implications. Studies point to the correlation between the burden of care and the risk of burnout, depression and its impact on the general health of caregivers [17]. As for the lack of specific training to deal with cardiovascular problems, it can be a major challenge for informal caregivers [18]. The need for educational and support resources is essential to improving the quality of care provided [19].

Despite their vital contribution, informal caregivers often lack institutional recognition. This gap highlights the need for policies that value and support their work [20].

They found that informal caregiving is significantly associated with poor health, suggesting that informal caregivers may experience negative consequences for their overall well-being [21]. Regarding the influence of caregiver burden on perceived health status among family caregivers of patients with chronic diseases, the results indicate that caregiver burden is significantly related to negative health perception [22].

A multicenter study conducted in Singapore yielded particularly striking findings: three months after a stroke, 63% of survivors still required informal care, and this figure only declined to 49% after one year. More remarkable was the increase in caregiving time: from an average of 64.3 h per week at three months to 76.6 h per week at twelve months. These results highlight not only the persistence of caregiving but also its progressive intensification over time — a burden that grows heavier rather than lighter as the months pass [23].

Multiple factors have been consistently linked to higher levels of perceived caregiver burden. One of the strongest predictors is the patient’s functional dependence: the greater the disability, the greater the need for continuous care, and consequently the higher the emotional and physical strain on caregivers. Another key factor is caregiver sex. Numerous studies have shown that women not only make up the majority of caregivers but also report higher levels of subjective burden, possibly due to the combined demands of caregiving, employment, and other traditionally assigned domestic responsibilities. The number of hours devoted to care is also relevant, although research suggests the relationship is not always linear — some caregivers, despite providing many hours of care, report lower levels of distress. Finally, the availability of additional support — for example, through paid domestic help — appears to buffer the perceived burden [24].

Informal caregiving after stroke is not merely a question of time or physical effort; it is a multidimensional experience that affects the caregiver physically, emotionally, socially, and economically. The scientific literature documents a wide range of challenges, from physical exhaustion and emotional stress to social isolation and sustained financial pressure [25].

It is estimated that approximately 27% of caregivers experience high levels of burden six months after a stroke. Poor quality of life, elevated stress levels, chronic emotional fatigue, and physical exhaustion emerge as clear risk indicators. Faced with this scenario, it is essential to move beyond merely describing the problem and to implement policies that include targeted educational programs, public awareness campaigns, and resources such as day-care units, which offer tangible relief for those who provide care day after day [26].

Nevertheless, despite the substantial body of evidence accumulated in recent years, important knowledge gaps remain. One of the most evident concerns the unequal attention given to caregiving depending on geographic region or cultural context. Most available studies originate from specific, often urban and resource-rich clinical settings, while multicenter or cross-national investigations that allow for the comparison of diverse realities and the exploration of cultural influences on the caregiving experience are still scarce.

Emerging psychosocial variables, such as resilience, meaning in caregiving, and perceived social support, are known to act as mediators between caregiver burden and quality of life, yet they have been insufficiently examined in depth. Their role, while promising, remains secondary in much of the current literature [17].

Another major challenge lies in moving from description to action. Although the factors that exacerbate caregiver burden are relatively well established, more robust findings are needed to design specific, context-sensitive interventions that are truly useful for caregivers. Observation alone is not enough; we must intervene.

Against this background, the present study has a clear objective: to assess the perceived burden among informal caregivers of stroke survivors and to determine the factors associated with that burden across four European countries, Portugal, Italy, Poland, and Spain. By incorporating clinical, sociodemographic, and psychosocial variables, this work provides a rare but necessary comparative multicenter approach. The ultimate goal is not only to expand current knowledge but also to generate solid evidence that can underpin targeted, culturally sensitive interventions and inform future policy and practice.

## Methods

### Study design and participants

The present study was conducted with a cross-sectional design involving 126 family caregivers of post stroke patients in four countries of Europe: Italy, Poland, Portugal and Spain. The sample size was defined based on methodological and practical criteria: it is an exploratory study to detect trends, so it was considered that approximately 30 participants per country were sufficient, in addition to ensuring balanced representation among the four countries.

Participants were recruited primarily through associations of relatives and patients of cardiovascular accidents (one association per country: Huelva, Spain; Florence, Italy; Warsaw, Poland; Lisbon, Portugal). Association leaders were contacted by telephone to request authorization to invite members to participate. After authorization, members attended an informative session explaining the study objectives and procedures and were invited to complete a web-based questionnaire via a QR code. To reach caregivers not affiliated with associations, participants were encouraged to share the survey link with others in similar caregiving situations, implementing a snowball sampling approach. The survey was conducted during February–March 2024.

### Measures

Data were collected via a self-administered online questionnaire comprising:

1. Demographic and caregiving information: age, gender, country/nationality, educational level, marital status, relationship with the care recipient, financial situation, health-related variables (use of psychotropic drugs, use of social and health care services), perception of physical and mental health, caregiving duration and weekly hours, voluntary caregiving, recognition of caregiver status, support received from family, friends, or public/non-profit organizations, and finding meaning in caregiving.
2. Caregiver burden: assessed using the Zarit Burden Interview (ZBI) [27]. Validated country-specific versions were used: Spanish [28], Portuguese [29], Italian [30], and Polish [31]. The ZBI consists of 22 items rated on a 5-point Likert scale (1 = never, 5 = almost always), with total scores ranging from 22 to 110; higher scores indicate greater burden. Scores were classified as absence (< 46), moderate (47–55), or severe (> 55) [20]. In this sample, Cronbach’s α = 0.903.

### Statistical analysis

Descriptive statistics were calculated: frequencies and percentages for categorical variables, means and standard deviations for continuous variables. Parametric tests were used following verification of assumptions: normality was assessed using the Kolmogorov–Smirnov test for the ZBI (D(118) = 0.78, *p* =.077), indicating no significant deviation; homogeneity of variances across factors was verified using Levene’s test (all *p* >.05).

Pearson correlations examined associations between ZBI scores and continuous variables. Differences between groups were assessed using Student’s t-tests or ANOVA, with post-hoc Tukey tests and adjusted p-values when comparing more than two groups. Effect sizes (Cohen’s d or η²) were reported where applicable. Statistical analyses were performed using SPSS version 27, with significance set at *p* <.05.

### Ethical considerations

Ethical principles such as avoiding harm to the participants, the right to withdraw from the study, the right to freely enter the research, and the confidentiality of information were observed in this study. The Ethics Committee of Atlantica University Institute (Lisbon, Portugal) granted approval for this study under the reference number 01 ESSATLA 2024.

## Results

### Sample characteristics

The final sample included 126 family caregivers of patients who have suffered a stroke (66% women, *n* = 83), distributed across countries: 25.4% Portuguese (*n* = 32); 24.6% Spanish (*n* = 31); 26.2% Italian (*n* = 33) and 23.8% Polish (*n* = 30). There were no significant differences in gender frequency between countries. Mean age was 57.8 years (SD = 13.2, range 18–84) with no differences across countries. Most participants had a university degree (56%) and a stable partner (80%), and 50% were employed. Regarding the caregiving relationship, 39% cared for a partner, 34% for a son/daughter, 12% for a parent, and 15% for others.

### Caregiver burden and descriptive data

This study showed that the average burden score on the ZBI was 56.6, representing an intense burden [22–46: absence of overload; 47–55: light overload; 56–88: intense overload], showing gender differences: women scored (x̄ = 58.73, *sd* 13.6) higher than men (x̄ = 52.11, *sd* 15.3) (t = 2.358; *p* =.020; *d*-Cohen 0.47); but no differences between countries (Italy x̄ = 58.71, *sd* 14.31; Poland x̄ = 57.37, *sd* 13.67; Portugal x̄ = 57.67, *sd* 15.46; and Spain x̄ = 52.69, *sd* 14.04).

Tables 1 and 2 summarize descriptive statistics for continuous and categorical variables, respectively.

**Table 1:** Mean scores and standard deviation

Variables (min-max)	Mean	Standard deviation
ZBI (22–110) [22–46: absence of overload; 47–55: light overload; 56–88: intense overload]	56.6	14.42
Physical health (1very good − 5 very bad)	2.35	0.81
Mental health (1very good − 5 very bad)	2.33	0.77
Take psychopharmacological medication (during last month) (1 daily – 4 never)	3.24	1.18
Use of social and health care services as a consequence of the care provided (during the last month) (1never – 4 more than 3 times)	1.77	0.92
Find meaning in caregiving (1 yes, always or most of the time – 3 No)	1.64	0.78
Quality of the relationship with the person they care for (1 very good – 5 very bad)	1.66	0.81

**Table 2:** Frequency and percentages

Variables		*N*	%
Officially recognised status as a caregiver	Yes	20	15.9
	No	104	82.5
Share the care	Yes	30	23.8
	No	74	58.7
Did you voluntarily and freely decide to take over the patient’s care?	Yes	26	20.6
	No	98	77.8
Receive financial support (for care) from institution	Yes	11	8.7
	No	113	89.7
Can call on volunteers or professional health and social workers in order to rest or to help when are unable to do so	Yes	22	17.5
	No	99	78.6
Can call on family or friends in order to rest or to help when are unable to do so	Yes	90	71.4
	No	32	25.4
When she/he began to care for	< 3 months ago	3	2.4
	3–6 months ago	2	1.6
	6–12 months ago	7	5.6
	1–2 years ago	19	15.2
	2–3 years ago	14	11.2
	3–4 years ago	21	16.8
	> 4 years ago	59	47.1
Weekly time spent on care	< 20 h	54	42.9
	20–40 h	9	7.1
	> 40 h	7	5.6
	All day, with ½ days to rest	9	7.1
	Virtually all day without rest days	45	35.7
Financial situation	Save every month	37	29.4
	It’s enough	71	56.3
	Don’t have enough to make ends meet	17	13.5
Take psychopharmacological medication (last month)	Daily	24	19
	Sometimes a week	4	3.2
	Less than once a week	16	12.7
	Never	82	65.1

### Associations between ZBI and other variables

As shown in Table 3, several variables were significantly correlated with the ZBI. These Pearson correlations indicate that higher levels of caregiver burden are associated with greater use of psychopharmacological medication, increased use of social and health care services, poorer self-rated physical and mental health, more hours of caregiving per week, lower quality of the relationship with the care recipient, reduced sense of meaning in caregiving, and worse financial situation. In summary, these results indicate that higher burden is linked to worse health, more caregiving demands, lower relational quality, and fewer psychosocial resources (Table 3).

**Table 3:** Correlations of ZBI score with study variables (*n* = 126)

Variables	r (ZBI)	*p*-value
Take psychopharmacological medication (during last month) (1 daily – 4 never)	− 0.230	0.12
Use of social and health care services as a consequence of the care provided (during the last month) (1never – 4 more than 3 times)	0.257	0.005
Physical health (1very good − 5 very bad)	0.366	< 0.001
Mental health (1 very good − 5 very bad)	0.537	< 0.001
Weekly time spent on care (min: <20 h; max: virtually all day without rest days)	0.261	0.004
Quality of the relationship with the person they care for (1 very good – 5 very bad)	0.408	< 0.001
Find meaning in caregiving (1 yes, always or most of the time – 3 No)	0.312	< 0.001
Financial situation (min: save every month; max: Don’t have enough to make ends meet)	0.333	< 0.001

### Group comparisons

The results of Student’s t-tests showed significant differences in the perceived burden depending on receive (or not) support from persons belonging to public, private or non-profit organizations (*t* = 2.669; *p* =.009, *d*-Cohen 0.66), and whether (or not) to perform care voluntarily (*t* = 3.693; *p* <.001, *d*-Cohen 0.83). As reported in Table 4, people who knew that they could count on the support of professionals or volunteers for the care of the patient scored lower on the burden of care than people who didn’t. Also, people who didn’t care voluntarily scored higher than people who did voluntarily.

**Table 4:** Mean and standard deviation on ZBI depending on different factors

Variables		Mean	Standard deviation
Receive support from persons belonging to public, private or non-profit organizations	Yes (*n* = 22)	49.00	14.46
	No (*n* = 99)	58.34	14.17
Did you voluntarily and freely decide to take over the patient’s care?	Yes (*n* = 26)	54.06	13.68
	No (*n* = 98)	65.44	13.56

No significant relationships were observed between burden and the official recognition of caregiver status, receiving public financial support, the length of time they have been practising care, sharing care with other family members or knowing that they can rely on them on an ad hoc basis.

## Discussion

This multicenter study, conducted across four European countries, provides a clear and well-substantiated picture of the burden borne by informal caregivers of stroke survivors. When analyzing the factors that influence this perceived burden, patterns emerge that are consistent with the most recent international literature [24, 26]. Indeed, the mean levels of burden, measured using the Zarit Burden Interview (ZBI), fall within a range that can be considered clinically concerning, reflecting a high level of caregiver strain. Far from being anecdotal, this finding aligns with previous studies reporting that between 25% and 54% of caregivers of stroke survivors experience high burden [25, 26]. Our sample, with a mean ZBI score of 56.6, shows figures similar to those reported in research conducted in both Europe and Asia [24, 32].

A particularly noteworthy finding was the difference by sex: women reported significantly higher burden levels than men, which is, unfortunately, unsurprising. The literature has long highlighted the disproportionate caregiving role assumed by women [33] — a phenomenon explained, at least in part, by factors such as traditional gender roles, the well-known “double workday” that combines paid employment with domestic care responsibilities, and deeply rooted cultural expectations. Moreover, recent research has emphasized that being female is, in itself, a risk factor for developing symptoms of depression, anxiety, and higher use of psychotropic medication [34].

Among the factors that contribute to intensifying caregiver burden, several well-known but still highly relevant ones stand out: poor physical and psychological health of the caregiver, many weekly hours devoted to care, strained or conflictive relationships with the care recipient, perceiving caregiving as meaningless, and experiencing persistent financial hardship. All of these have been widely documented in previous studies [35].

Despite this challenging reality, there is room for hope. Our findings indicate that when caregiving is performed voluntarily — rather than out of obligation — and when there is some form of institutional or community support network, perceived burden decreases significantly. This observation reinforces what other studies have shown: the value of social support and personal resilience as buffers against the emotional impact of caregiving [36, 37].

Finally, we found a clear association between higher caregiver burden, greater use of psychotropic medications, and more frequent use of healthcare services. Although this does not imply a direct causal relationship, it reveals a worrying pattern: caregiving without rest and without adequate support may deteriorate the caregiver’s mental health, leading to pharmacological dependence and increasing pressure on healthcare systems. This dynamic is consistent with the findings of several recent meta-analyses [38] and systematic reviews [39] that directly link prolonged caregiver burden with symptoms of anxiety, stress, and affective disorders. In line with this, qualitative research has highlighted a frequently overlooked aspect: when caregivers are able to find meaning in their role — when caregiving connects with something personally valuable — the experience ceases to be solely a source of strain and becomes, at least in part, an act of resilience [40].

The idea referred to by Marino et al., that care is written in the feminine and singular [41], is contrasted again in our study, taking into account that the distribution of participants according to the countries was similar − 24.6% Portuguese; 24.6% Spaniards; 23.8% Italians and 23% Poles – two-thirds of these were women, with no significant differences in sex frequency between these countries. However, sex is significant in the overload variable, this being one of the most remarkable results of our research.

Taking into account the caregiver’s mental health described above, there are different studies [42, 43] that coincide with our results, significantly associating caregiver overload with anxiety and the consumption of psychotropic drugs, which is statistically relevant in each of the countries surveyed. A meta-analysis developed in different European universities [44] synthesized the evidence related to subjective caregiver burden and anxiety symptoms in informal caregivers, concluding a large positive association between subjective caregiver burden and anxiety symptoms (*r* =.51; 95% CI = 0.47, 0.54; I 2 = 0.0%). This may explain the association between informal caregivers and psychotropic drug use as shown in our study. A study carried out by an international team from Spain, Portugal and the United States agrees with this result, stating that being a woman, older and dedicating more hours per week to caring for family and friends has a higher risk of depression, anxiety and use of antidepressants or anxiolytics [34]. This requires a preventive approach based on intersectoral public-private collaboration for the co-design of health and social care services, especially non-pharmacological approaches [45].

The correlational analyses in this study highlighted a series of factors that act as true amplifiers of caregiver burden. Among the most influential are the deterioration of the caregiver’s physical and psychological health, the high number of weekly hours dedicated to care, a strained or conflictual relationship with the care recipient, the perception of caregiving as meaningless, and, of course, persistent financial hardship. In other words, it is not enough to know how much care is provided; it is equally important to understand the emotional, relational, and material conditions under which that caregiving takes place.

These results are consistent with the findings of Giray and colleagues, who noted that patient functional disability combined with the caregiver’s low socioeconomic status creates a particularly vulnerable scenario, where multiple risk factors intertwine and reinforce each other in a vicious cycle of strain that rarely resolves on its own [35].

Nonetheless, the study also offers a ray of hope. We observed that when caregiving is undertaken voluntarily and there is some form of institutional or community support, perceived burden tends to decrease. This does not eliminate the inherent effort required for caregiving, but it makes the experience more manageable and more shared. This observation resonates with previous research underscoring the value of community support and resilience as key elements in sustaining caregivers’ emotional balance [36, 37].

Furthermore, we identified a clear correlation between higher levels of caregiver burden, increased consumption of psychotropic medication, and more frequent use of healthcare services. While this association does not establish direct causality, it outlines a concerning pattern: when caregiving becomes chronic and there are no opportunities for respite or support, caregivers’ mental health begins to deteriorate. In many cases, this leads to pharmacological dependence and an increasing demand for healthcare resources.

This pattern is consistent with the meta-analysis by Kaddour and Kishita, which demonstrated a robust association between subjective caregiver burden and anxiety symptoms [38]. Similarly, it aligns with the systematic review by Del-Pino-Casado and colleagues, which directly links sustained caregiver burden with the onset of affective disorders [39]. Together, these studies underscore the urgent need for preventive strategies that not only alleviate caregiver burden but also protect the emotional well-being of those who provide care.

Another consequence of caregiver overload highlighted in our research, which in turn is related to the finding described above, is the hyper-frequentation of social and health services by informal caregivers, which coincides with another study related to the same population, caregivers of cardiovascular patients at Suzhou Medical College of Soochow University [46].

This study describes an association between institutional social support and levels of overload, understanding that greater perceived institutional social support is related to lower levels of burden, the same occurs in various studies of informal caregivers [47]. Our results also support that the fact of exercising care not by express voluntariness, but by family and cultural pressure makes the feeling of overload greater than in those who do it voluntarily being socially supported, so it is necessary to plan interventions aimed at promoting social support [48].

Another of the studies consulted coincides with our research [49], it showed that improving caregivers’ health literacy effectively improves their care capacity, in addition to affirming that social support is capable of linking health literacy and care capacity.

It is worth highlighting a significant finding in our study related to the meaning that our respondents have regarding the care of their relatives. The greater the sense of care with their voluntariness, the less overload they feel. A qualitative study carried out at Nottingham Trent University [50], reported that the meaning that caregivers associate through care motivated them to face the difficulties associated with caring for a person in need. This research found that most informal caregivers perform it based on cultural conceptions of role and gender that make it obligatory for them, so they require support to reduce the pressures associated with caregiving and allow them to continue to lead a meaningful life both inside and outside their role as caregivers. To provide real support to these caregivers and to ensure that they are not alone in this care work, health services need to be strengthened by expanding the services provided by multiprofessional teams in all health and social settings, especially in the homes of patients and caregivers [51].

Despite the merits of this multicenter study and the important findings that contribute to improving care for family caregivers of people with CVD, it also has limitations.

A key limitation of this study relates to the measurement of certain variables, particularly physical and mental health, which were assessed using a single-item measure. While this approach allowed for a rapid and feasible assessment in a multicenter study, it may have limited the sensitivity and precision with which these dimensions were captured, potentially underestimating subtle variations in caregiver well-being. Similarly, other psychosocial variables were self-reported, which may introduce recall bias or social desirability effects.

Another important limitation is the cross-sectional design, which restricts interpretation to associations and does not permit any inference of temporal sequence or causality. This is particularly relevant given that caregiver burden is a dynamic phenomenon, influenced by both the clinical trajectory of the stroke survivor and changes in the caregiver’s personal, social, and economic circumstances over time.

Future research should therefore prioritize longitudinal designs, enabling the monitoring of caregiver burden across the different phases of stroke recovery and capturing how psychosocial, relational, and clinical factors interact dynamically. Studies of this nature should also employ validated, multidimensional instruments to assess caregivers’ physical and mental health, incorporate measures of resilience and quality of the caregiver–recipient relationship, and systematically collect clinical data on the care recipients — including functional status, comorbidities, time since stroke, and severity of disability. Integrating these dimensions would provide a more comprehensive and nuanced understanding of caregiver burden and could inform the design of targeted interventions and policies that are culturally sensitive and tailored to caregivers’ specific needs at different stages of the caregiving trajectory.

## Conclusions

This study provides a deep and up-to-date insight into the reality of informal caregivers of stroke survivors across four European countries. Far from being a homogeneous experience, caregiving emerges as a complex phenomenon that spans multiple dimensions — physical, emotional, social, and economic. Our results make visible a burden that is not only intense but also persistent, particularly in the absence of adequate support and sufficient recognition.

We identified factors that exacerbate this burden — such as poor caregiver health, a deteriorated relationship with the care recipient, and a lack of perceived meaning in the caregiving role — as well as others that act as buffers, including voluntariness in assuming the role, institutional support, and community networks. This dual perspective not only describes the phenomenon but also points to possible avenues for intervention.

Moreover, we observed a concerning association between high levels of caregiver burden, greater use of psychotropic medication, and more frequent reliance on healthcare services. This relationship calls for reflection not only on the consequences of prolonged, unsupported caregiving but also on the silent ways in which health systems shift the weight of care onto families, often without providing them with a real safety net.

Despite the abundant existing literature, important gaps remain. On one hand, the lack of multicenter studies with a comparative approach limits the development of coordinated strategies at the European level. On the other, psychosocial variables such as meaning-making, resilience, and the quality of the caregiver–patient relationship remain underexplored despite their clear impact. This study thus provides a solid foundation on which to build further knowledge that is both useful and applicable, always keeping the focus on the well-being of those who sustain the invisible backbone of our health systems: informal caregivers.

Ultimately, caregiving should neither be a source of chronic exhaustion nor of invisibility. Promoting public policies that are sensitive to this reality, designing emotional support programs, facilitating access to resources, and training health professionals to address informal caregiving are essential steps toward a fairer, more humane, and more sustainable model of care.

The role of caregiver described a woman who cares alone, with low economic levels and care overload, faces greater difficulties than other caregivers, dedicating more time to the established care tasks, losing job opportunities that would allow them to achieve better care and working conditions and a better quality of life.

These findings underscore the urgent need to design and implement targeted interventions that foster resilience and meaning in caregiving, develop psychosocial and educational programs, and promote public policies that ensure adequate health and social resources for caregivers. Nevertheless, the results should be interpreted with caution. Future research employing longitudinal designs and more comprehensive data collection, including information on both caregivers and care recipients, will be essential to better understand how caregiver burden evolves over time and to evaluate the effectiveness of interventions tailored to different cultural contexts.

## Supplementary Information

Supplementary Material 1.

## Data Availability

The datasets used and/or analysed during the current study are available from the corresponding author on reasonable request.
