# Supplementary material for: Factors that influence the burden of the caregiver of cardiovascular patients. A multicenter study
Source: BMC Public Health. 2025 Nov 21;25:4098. doi: 10.1186/s12889-025-25074-0 (PMC12639714; doi:10.1186/s12889-025-25074-0)
Supplement: Supplementary file 1 — Supplementary Material 1. [file 12889_2025_25074_MOESM1_ESM.pdf]

# QUESTIONNAIRE_English version

**Informed Consent**

You are invited to participate voluntarily in a survey on informal caregivers conducted by the Universities of Atlantica (Portugal), Firenze (Italy), Huelva (Spain) and the Warsaw School of Economics (Poland) and founded by HaDea. This survey aims to collect data on several important topics related to the care of people with CVD (cardiovascular disease) to identify possible needs. The survey will ask for your opinion, personal experiences, and knowledge, among other things. Your responses will be used solely for the purposes previously discussed and will be treated with absolute confidentiality. Your participation in this study will have no personal consequences or repercussions.
There are no right or wrong answers, we simply seek your honest opinion and experience. You may choose to withdraw from the study at any time without any negative impact on yourself.

If you wish to participate in this study, please indicate your agreement by ticking the box marked 'I agree' after reading the following consent form:

"I have been informed about the Informal Caregivers study. I hereby give my consent to participate in this study and allow the research team to analyze and publish the information obtained from me, anonymously and together with the information obtained from the other participants, for scientific and applied purposes".

- o I'm over 18 and I agree
- o I disagree

**I. About the person you care for**

1. When did you begin to care for the patient?
    1. Less than 3 months ago
    2. Between 3 to 6 months ago
    3. Between 6 months to a year ago
    4. Between 1 and 2 years ago
    5. Between 2 or 3 years ago
    6. 3 to 4 years ago
    7. More than 4 years ago

2. Did you voluntarily and freely decide to take over the patient's care?
    1. No, I did it because there was no other option.
    2. Yes, I did.

3. How many hours per week do you spend as a caregiver?
    1. Less than 20 hours per week (i.e. 4 hours per day for 5 days per week).
    2. 20 to 40 hours per week (i.e. 8 hours per day for 5 days per week).
    3. More than 40 hours per week.
    4. Practically all day, with 1 or 2 days off per week.
    5. Virtually all day, with no rest days at all.

4. With whom do you share patient care and attention? You may select multiple options:

1. No one
2. With a family member
3. With a neighbour or friend
4. With a hired person(s)
5. With several persons (others not mentioned):__________________

5. In general, how would you define your relationship with the patient?

1. Very good
2. Good
3. Neither good nor bad
4. Bad
5. Very bad

6. Do you have officially recognised status as a caregiver?

1. Yes
2. No

7. Do you receive any financial support (for care) from any institution?

1. Yes
2. No

8. Do you feel that you have found meaning in the care you provide to the patient?

1. Yes, always or most of the time
2. Yes, although sometimes I have doubts
3. No

**II. Zarit Caregiver Burden Interview (ZBI)**

Instructions: The following is a list of statements that reflect how people sometimes feel when taking care of another person. After reading each statement, indicate how often you experience the feelings listed by choosing the number that best corresponds to the frequency of these feelings. Remember that there are no right or wrong answers.

9. Do you feel stressed between caring for your relative and trying to meet other responsibilities for your family or work?

(1) Never (2)Almost Never (3)Sometimes (4)Frequently (5)Nearly Always

10. Do you feel embarrassed you're your relative's behavior?

(1) Never (2)Almost Never (3)Sometimes (4)Frequently (5)Nearly Always

11. Do you feel angry when you are around your relative?

(1) Never (2)Almost Never (3)Sometimes (4)Frequently (5)Nearly Always

12. Do you feel that your relative currently affects your relationship with other family members or friends in a negative way?

(1) Never (2)Almost Never (3)Sometimes (4)Frequently (5)Nearly Always

13. Are you afraid what the future holds for your relative?

(1) Never (2)Almost Never (3)Sometimes (4)Frequently (5)Nearly Always

14. Do you feel strained when you are around your relative?
(1) Never (2)Almost Never (3)Sometimes (4)Frequently (5)Nearly Always

15. Do you feel that you do not have as much privacy as you would like because of your relative?
(1) Never (2)Almost Never (3)Sometimes (4)Frequently (5)Nearly Always

16. Do you feel that your social life has suffered because you are caring for your relative?
(1) Never (2)Almost Never (3)Sometimes (4)Frequently (5)Nearly Always

17. Do you feel uncomfortable about having 0 friends over because of your relative?
(1) Never (2)Almost Never (3)Sometimes (4)Frequently (5)Nearly Always

18. Do you feel that you have lost control 0 of your life since your relative's illness?
(1) Never (2)Almost Never (3)Sometimes (4)Frequently (5)Nearly Always

19. Do you wish you could just leave the 0 care of your relative to someone else?
(1) Never (2)Almost Never (3)Sometimes (4)Frequently (5)Nearly Always

20. Do you feel uncertain about what to do about your relative?
(1) Never (2)Almost Never (3)Sometimes (4)Frequently (5)Nearly Always

21. Do you feel that you should be doing more for your relative?
(1) Never (2)Almost Never (3)Sometimes (4)Frequently (5)Nearly Always

22. Do you feel you could do a better job in caring for your relative?
(1) Never (2)Almost Never (3)Sometimes (4)Frequently (5)Nearly Always

23. How burdened do you feel in caring for your relative?
(1) Never (2)Almost Never (3)Sometimes (4)Frequently (5)Nearly Always

24. Do you feel that your relative asks for more help than (s)he needs?
(1) Never (2)Almost Never (3)Sometimes (4)Frequently (5)Nearly Always

25. Do you feel that because of the time you spend with your relative that you do not have enough time for yourself?
(1) Never (2)Almost Never (3)Sometimes (4)Frequently (5)Nearly Always

26. Do you feel your relative is dependent upon you?
(1) Never (2)Almost Never (3)Sometimes (4)Frequently (5)Nearly Always

27. Do you feel your health has suffered because of your involvement with your relative?
(1) Never (2)Almost Never (3)Sometimes (4)Frequently (5)Nearly Always

28. Do you feel that your relative seems to expect you to take care of him/her as if you were the only one he/she could depend on?
(1) Never (2)Almost Never (3)Sometimes (4)Frequently (5)Nearly Always

29. Do you feel that you will be unable to take care of your relative much longer?

(1) Never (2)Almost Never (3)Sometimes (4)Frequently (5)Nearly Always

30. Do you feel that you do not have enough money to care for your relative in addition to the rest of your expenses?

(1) Never (2)Almost Never (3)Sometimes (4)Frequently (5)Nearly Always

**III. State of health of the caregiver**

31. In general, how would you define your state of physical health?

1. Very good
2. Good
3. Not so good
4. Bad
5. Very bad

32. In general, how would you define your state of psychological/mental health?

1. Very good
2. Good
3. Not so good
4. Bad
5. Very bad

33. During the last month, have you required social-health care as a consequence of your patient's care (i.e. have you been to a doctor, nurse, social worker, physiotherapist, rehabilitation, psychologist, psychiatrist, etc.?)?

1. Never
2. Once
3. Two or three times
4. More than three times

34. Can you call on family or friends to help you care for your patient in order to rest or when you are unable to do so yourself?

1. Yes
2. No

35. Can you call on volunteers (i.e. from associations, NGO…) or professional health and social workers (from health services) to help you care for your patient in order to rest or when you alone are unable to do so?

1. Yes
2. No

36. Do you currently (during the last month) take any kind of psychopharmacological medication (anxiolytics, antidepressants, sleep inducers, etc.)?

1. Yes, daily
2. Yes, once or several times a week
3. Yes, occasionally (less than once a week)
4. No

**IV. Socio-demographic questions (of the caregiver)**

37. Gender:

1. Female
2. Male
3. Other

38. Age (years, please write in numbers, i.e. 67): _______________

39. Highest level of education attained:

1. None
2. Primary school
3. Secondary school
4. Post-Secondary School Professional Training. Which one? _____________
5. Advanced vocational training studies. Which one? _____________
6. Bachelor’s degree. Which one? _____________
7. Master’s degree. Which one? _____________
8. PhD. Which one? _____________
9.

40. Marital status:

1. Without a stable partner
2. With stable partner – living together
3. With stable partner – not cohabiting
4. Widowed
5. Separated or divorced
6. Other: ___________

41. Number of dependants: 1 / 2 / 3/ 4 / 5 / 6 / more than 6

42. Thinking about the income of the entire household (counting all the income of the people living in your household), Which of the following best describes your financial situation?

1. I save every month
2. It’s enough
3. I don’t have enough money to make ends meet
